# *In Vitro* and *In Vivo* Characterizations of Chiglitazar, a Newly Identified PPAR Pan-Agonist

**DOI:** 10.1155/2012/546548

**Published:** 2012-10-22

**Authors:** B. K. He, Z. Q. Ning, Z. B. Li, S. Shan, D. S. Pan, B. C. B. Ko, P. P. Li, Z. F. Shen, G. F. Dou, B. L. Zhang, X. P. Lu, Y. Gao

**Affiliations:** ^1^Exploratory Research Department, Shenzhen Chipscreen Biosciences Ltd., BIO-Incubator, Suite 2-601, Shenzhen Hi-Tech Industrial Park, Guangdong, Shenzhen 518057, China; ^2^Department of Pharmacology and Toxicology, Beijing Institute of Radiation Medicine, 27 Taiping Road, Beijing 100850, China; ^3^Department of Chemistry, The University of Hong Kong, Hong Kong; ^4^Institute of Materia Medica, Chinese Academy of Medical Sciences & Peking Union Medical College, Beijing, China; ^5^Laboratory of Drug Metabolism and Pharmacokinetics, Beijing Institute of Transfusion Medicine, Beijing, China; ^6^Tianjin University of Traditional Chinese Medicine, Tianjin, China

## Abstract

Solid rationales are still present for the identification of synthetic ligands to simultaneously target multiple PPAR subtypes for the treatment of T2DM. The purpose of this study was to characterize the *in vitro* and *in vivo* differential effects of chiglitazar, a non-TZD type of PPAR pan-agonist currently in phase III clinic development in China, from PPAR**γ**-selective agonist like rosiglitazone. Chiglitazar showed transactivating activity in each PPAR**α**, **γ**, and **δ** subtype and upregulated the expression of PPAR**α** and/or PPAR**δ** downstream genes involved in the key processes of lipid metabolism and thermogenesis. Comparable blood glucose lowering effect was observed between chiglitazar and rosiglitazone, but chiglitazar did not significantly increase the body weight in KKAy and fat pad weight in *db/db* mice. Chiglitazar had high distribution in liver, pancreas, and skeleton muscles but was less present in kidney, heart, and adipose in rats. Heart weight increase was not observed in rats treated with chiglitazar for 6 months at a dose as high as 45 mg kg^−1^. The *in vitro* and *in vivo* differential features of chiglitazar are informative and encouraging for the further development of this synthetic ligand for the potential use in T2DM.

## 1. Introduction

Peroxisome proliferator-activated receptors (PPARs) are ligand-activated transcription factors that belong to the nuclear hormone receptor superfamily. These receptors, consisting of PPAR*α*, PPAR*γ*, and PPAR*δ*, form heterodimers with the retinoid X receptor (RXR), bind to specific DNA sequences in the regulatory region of target genes, and modulate their transcription. PPARs initially became the focus of intense investigation following discoveries that PPAR*α* and PPAR*γ* are the molecular targets of major classes of drugs. Fibrates, effective in the treatment of hyperlipidemia, target PPAR*α*, while thiazolidinediones (TZDs) are high-affinity ligands for PPAR*γ* that have revolutionized the treatment of type 2 diabetes mellitus (T2DM) by directly alleviating tissue insulin resistance, the central mechanism underling the onset and development of T2DM [1].

PPAR*γ* is predominantly present in adipose tissue and functions as a master regulator of adipocyte differentiation and metabolism [2]. It is generally accepted that by binding and activating PPAR*γ*, TZDs repartition fatty acids (FAs) to adipose tissue and away from muscles, liver, and circulation, thus improving insulin resistance [3]. PPAR*α* is highly expressed in liver, skeletal muscles, heart, and cells of atherosclerotic lesions. Acting as a molecular sensor of endogenous FAs and their derivatives, PPAR*α* is an important regulator of fatty acid metabolism and energy homeostasis. PPAR*α* also exerts pleiotropic antiinflammatory and antiproliferative effects and prevents the proatherogenic effects of cholesterol accumulation in macrophages by stimulating cholesterol efflux [4]. PPAR*α*-mediated hypolipidemic and vascular effects of fibrates may contribute to a reduction of cardiovascular events and may explain their clinical benefits observed in human trials [5]. PPAR*δ* has the broadest expression pattern and is less well characterized. Receptor knockouts revealed multiple developmental and homeostatic abnormalities in PPAR*δ*-null mice, indicating it as a key regulator with the potential to therapeutically target multiple aspects of the metabolic syndrome [6]. In animal models, PPAR*δ* agonists retarded weight increase under high-fat diet conditions while maintaining insulin sensitivity, probably by stimulating skeletal muscle fatty acid metabolism and thermogenesis [7]. Recent results from humans with a PPAR*δ* selective agonist have shown the reversal of multiple metabolic abnormalities in obese men [8]. 

Given the overlapping but differential biological functions of each PPAR subtype in the regulation of lipid and glucose metabolism and associated energy homeostasis, identification of novel synthetic ligands that simultaneously target multiple PPAR subtypes for T2DM and associated complications has been heavily pursued. However, such attempts were overshadowed by the terminations of several PPAR*α*/*γ* dual agonists in clinical development, apparently due to safety concerns. For example, muraglitazar showed superior efficacy in both hyperglycemia and dyslipidemia control in patients with T2DM [9], but its cardiovascular safety raised concerns [10] which prompted the discontinuation of further development from the sponsor [11]. However, in the case of muraglitazar, according to publicly accessible data [9], the adverse effects in humans appear to be linked only with the known PPAR*γ* activation-mediated class effects, such as water retention, increased adiposity, and the resulting body weight increase [12, 13], as well as cardiovascular safety concerns that have also been raised recently about rosiglitazone [14], a TZD type of PPAR*γ*-selective agonist. However, other recently developed non-TZD chemicals that are defined as partial agonists or selective PPAR*γ* modulators (SPPARMs) possess comparable effect on plasma glucose lowering and insulin sensitization; meanwhile lack of edema and weight gaining commonly related to TZD-type agonists, providing solid evidences to apportion efficacy from undesired effects via rational chemical design [15–17]. Whilst there is less doubt that synthetic ligands with multiple subtype activity improve overall efficacy in T2DM, particularly in associated dyslipidemia, there is still a need to accumulate experimental knowledge, particularly in humans, about whether the addition of *α* and/or *δ* activity for a ligand along with *γ* activity would cause additive or more mechanism-mediated adverse effects, or whether the additional *α*/*δ* activity might actually contradict the *γ*-activation-mediated unwanted effects, thus leading to a synthetic ligand with an overall better profile in patients with T2DM.

Chiglitazar has previously been reported as a PPAR*α*/*γ* dual agonist that significantly increases insulin sensitivity and has, compared with rosiglitazone, a much improved lipid profile in MSG- (monosodium L-glutamate-) induced obese rats [18]. In the current study, we have further performed *in vitro* and *in vivo* experiments to characterize chiglitazar as a PPAR pan-agonist. Differential effects on gene regulation and an overall better *in vivo* profile of chiglitazar compared with rosiglitazone have been demonstrated, consistent with the mechanism of action of chiglitazar and its favorable tissue distribution pattern. 

## 2. Methods

### 2.1. Chemicals

Chiglitazar (2-[(2-(4-fluorobenzoyl)phenyl)amino]-3-[4-(2-carbazolylethoxy)-phenyl]-propionic acid) was discovered and synthesized by Chipscreen Biosciences Ltd., whose chemical structure has been shown in the previous report [18]. Rosiglitazone and pioglitazone were provided by Jiangsu Depei Chemical Co. Ltd. (Jintan, China). WY14643 and 2-bromohexadecanoic acid were purchased from BIOMOL International (Plymouth Meeting, PA, USA). The purity for all chemicals was over 98.0% by HPLC analysis. 

### 2.2. Plasmids and Cell Lines

cDNAs for human retinoid X receptor (hRXR), PPAR*α*, PPAR*γ*, and PPAR*δ* were obtained by reverse transcriptase polymerase chain reaction (RT-PCR) from human liver or adipose tissues. Amplified cDNAs were cloned into pcDNA3.1 expression vector (Invitrogen, Carlsbad, CA, USA). The integrity and fidelity of all constructs made were verified by DNA sequencing. Luciferase reporter plasmids, ACOX promoter for PPAR*γ* and pHD(X3)-luc for PPAR*α*, were kind gifts of Drs. TM McIntyre (University of Utah, USA) and RA Rachubinski (University of Alberta, Canada), respectively. PPRE-luc for PPAR*δ* was constructed based on the previous report [19] by insertion of the annealed oligonucleotides having four copies of the CYP4A6 PPRE (4 × AGGTCAAAGGTCA) into the upstream of the luciferase coding sequence in pGL3-promoter vector (Promega, Madison, WI, USA). pCMV*β*Gal was purchased from Clontech (Palo Alto, CA, USA). The mouse 3T3-L1 preadipocyte cell line, skeletal muscle C2C12 cell line, and macrophage RAW267.4 cell line and the human osteosarcoma U2OS cell line were obtained from the American Type Culture Collection (ATCC, Rockville, MD, USA). The human hepatoma SMMC-7721 cell line was obtained from the Cell Culture Center of Chinese Academy of Medical Sciences (Beijing, China). Cell lines were cultured in medium of DMEM (3T3-L1, C2C12, and SMMC-7721), RPMI 1640 (RAW267.4), or McCoy's 5A (U2OS), containing 10% fetal bovine serum (FBS), 50 *μ*g/mL streptomycin, and 50 units/mL penicillin at 37°C in a humidified incubator with 5% CO_2_.

### 2.3. Transactivation Analysis by Reporter Gene Assays

SMMC-7721 or U2OS cells were seeded in 96-well plates the day before transfection to give a confluence of 50–80% when transfection was performed. A total of 60 ng of DNA containing 10 ng of hRXR, 10 ng of pCMV-*β*-Gal, 10 ng of expression vector containing each PPAR subtype, and 30 ng of the corresponding reporter plasmid was cotransfected per well using FuGene6 transfection reagent (Roche Molecular Biochemicals, Indianapolis, IN, USA) according to the manufacturer's instructions. 24 h after transfection, cells were incubated with Dulbecco's modified Eagle's medium containing 10% charcoal-stripped FBS and were treated with the individual compounds dissolved in dimethylsulfoxide (DMSO) for 24 h. The final concentration of DMSO in culture medium was 0.1%. Cells were lysed and prepared for the measurement of luciferase activity using a luciferase assay kit from Promega. Luciferase enzyme activity was detected by the Ascent Fluoroskan FL reader (Thermo Labsystems, Helsinki, Finland). To measure *β*-galactosidase activity, 50 *μ*L of supernatant from each transfection lysate was transferred to a new microplate, and the enzyme activity was detected by a reagent kit from Promega and read in a microplate reader (Bio-tek Instruments Inc., Winooski, VT, USA). The *β*-galactosidase data were used to normalize the luciferase data. The serial dilution for each tested compound was made, and 9 concentrations were used to generate the response curve with highest concentration set at 100 *μ*M (Wy-14643) or 20 *μ*M (Rosiglitazone, 2-Bromohexadecanoic acid, Chiglitazar), respectively. The transactivity was measured with reporter gene assay, and EC_50_ values for each compound were determined against their concentration-response curves.

### 2.4. Gene Expression Analysis by RT-PCR

Total RNA was extracted from cells or tissues with an RNeasy minikit (Qiagen, Valencia, CA, USA). The first-strand cDNA was synthesized using the oligo(dT) primers, followed by using SuperScript II reverse transcriptase (Invitrogen) according to the manufacturer's instructions. The PCR amplifications were performed in 10 *μ*L of reaction mixture volume containing 0.8 *μ*L of cDNA, 10 mM Tris-HCl (pH 8.3), 50 mM KCl, 1.5 mM MgCl_2_, 0.1% Triton X-100, 0.2 mM deoxynucleotide triphosphates, 0.4 *μ*M primers, and 0.25 U of Taq polymerase (TaKaRa Biotechnology Co., Ltd, China). Primers and the sequences for PCR are shown in Table 1. Taq DNA polymerase was added to each tube after a treatment of 2 min at 94°C. This was followed by 22–30 cycles of denaturation (23 s at 94°C), annealing (20 s at 55°C), extension (30 s at 72°C), and a final extension step of 2 min. PCR products were analyzed by electrophoresis on a 1.5% agarose gel stained with ethidium bromide.

### 2.5. Diabetic Mice and Treatment

All animals were maintained at the controlled temperature (22 ± 1°C), under 12 h light/dark cycles, and given standard laboratory chow and water *ad libitum*. Protocols for animal experiments were in accordance with the National Institutes of Health guidelines.

Male *db/db* mice (BKS.Cg-m +/+ LEPR^*db*^) in 6-7 weeks were purchased from The Jackson Laboratory (Bar Harbor, ME, USA). Male KKAy and C57BL/6J mice in 8–10 weeks were obtained from the Institute of Animal Science, Chinese Academy of Medical Sciences and Peking Union Medical College (Beijing, China). Animals were randomly sorted into different treatment groups (8–10 per group). From the next day, different groups of KKAy or *db/db* mice were orally treated daily with chiglitazar suspended in water at the doses of 5, 10, and 20 mg kg^−1^ (5 and 20 mg kg^−1^ for KKAy mice), rosiglitazone at 5 mg kg^−1^, and vehicle (water), respectively, for 12 (KKAy) and 14 (*db/db*) days. Plasma glucose levels were examined at various time points by Accu-Chek Advantage II Glucose Strips (Roche Diagnostics, Basel, Switzerland) and measured by Accu-Chek Advantage (Roche Diagnostics). Animals were sacrificed 24 h after the last dosing and abdominal fat pad, liver, and skeletal muscle from KKAy mice and abdominal fat pad from *db/db* mice were taken for gene expression analysis. 

## 3. Glucose Tolerance Test and Plasma Insulin Measurement

For intraperitoneal glucose tolerance test (IPGTT), *db/db* mice dosed for 14 days were fasted overnight, followed by an intraperitoneal injection of glucose (2 g kg^−1^). Blood samples were taken for measurement of plasma glucose before and 30, 60, and 120 min after the glucose load. The area under curves (AUC) was calculated to evaluate the IPGTT results. To determine plasma insulin concentrations, blood samples were taken from *db/db* mice dosed with different reagents or vehicle for 0, 3, 6, and 9 d, and insulin levels were measured by Insulin ELISA Kit (Roche Diagnostics) following the instructions from the manufacture. 

### 3.1. Influence in Heart Weight of Rats

Male and female Wistar rats of 6-week old were randomly divided into 4 groups, 5 for each male and female per group. Animals in different groups were orally treated daily with chiglitazar suspended in water at the doses of 15, 45, and 135 mg kg^−1^, or vehicle (water), for 6 months. 24 h after the last treatment, animals were sacrificed and the weight of heart from individual animals was weighed. 

### 3.2. Tissue Distribution in Rats

^3^H-chiglitazar was synthesized and provided by the Beijing Institute of Atom Energy with specific activity of 15 Ci mmol^−1^ and purity >97%. Tissue distribution of ^3^H-chiglitazar in rats was evaluated based on the methods described previously [20]. Briefly, male Wistar rats (~220 g in body weight, 12-week old) were randomly divided into 4 groups (5 per group). After fasted overnight, rats were orally administered with single dose of 12.5 mg kg^−1^ of  ^3^H-chiglitazar (radiation dose 31.7 MBq kg^−1^). Rats in different groups were sacrificed at 1.5, 4, 8, and 24 h after dosing. Blood (5–9 mL) was collected via cardiac puncture at the time of sacrifice. Liver, stomach, small intestine, large intestine, pancreas, brain, heart, lung, kidney, spleen, skeletal muscle, skeleton, abdominal fat, adrenal gland, bladder, and testis were removed from each animal by gross dissection. Tissues were rinsed gently, but thoroughly, with saline to remove remaining traces of blood before storage. Dissecting instruments were also washed between tissue procurements to avoid crosscontamination. Tissue suspensions were made with distilled water and centrifuged at 14,000 g for 5 min. 100 *μ*L of supernatant from each sample was taken and replaced in 24 well plates. 1 mL of scintillation fluid was added to each well, and radioactivity was measured by a scintillation counter (Perkin Elmer, Waltham, MA, USA). 

### 3.3. Statistical Analysis

Nonlinear regression analysis was used to determine the EC_50_ and ED_50_/ED_25_ of the synthetic ligands in transactivation assays and blood glucose-lowering effect in *db/db* mice, respectively. Data are expressed as mean ± SE. The significance of differences was analyzed by using a Student's *t*-test.

## 4. Results

### 4.1. *In Vitro* Activity and Regulation of Gene Expression

**Figure 1:**
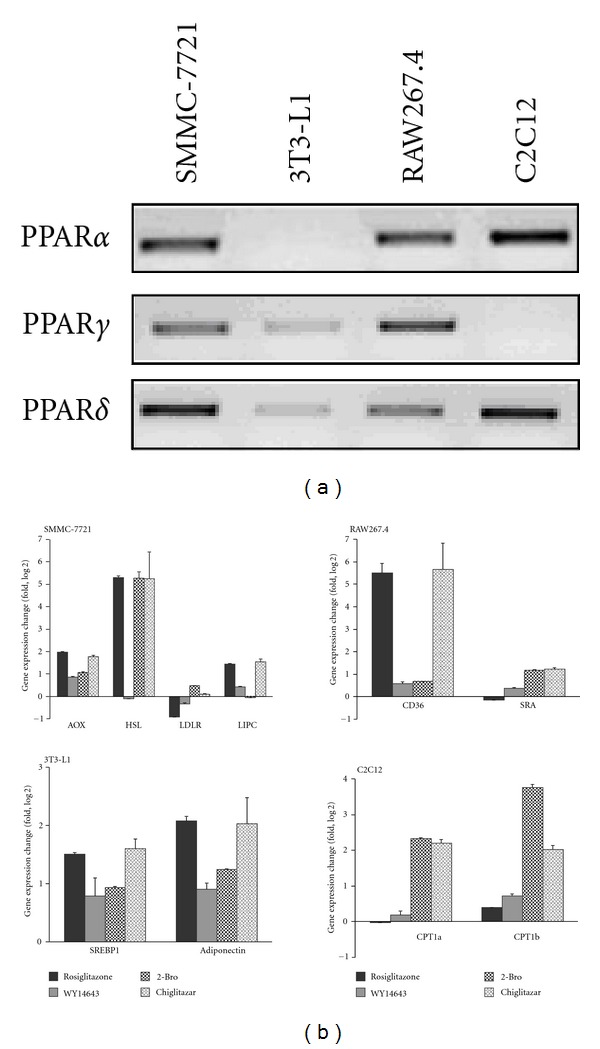
*In vitro* regulation of gene expression by chiglitazar. (a) Endogenous expression of individual PPAR subtypes in cell lines. (b) The indicated cell lines were treated with 10 *μ*M rosiglitazone, 50 *μ*M WY14643, 5 *μ*M 2-bromohexadecanoic acid (2-Bro), 10 *μ*M chiglitazar, or vehicle control (0.1% DMSO), respectively, for 48 h. Total RNA was isolated and RT-PCR was carried out using primers of the individual genes as listed in Table 1. The expression change of referred genes induced by different compounds was normalized against internal control (36B4 or GAPDH) and then compared with vehicle control treatment. The data shown are averaged from 3 independent experiments.

To evaluate the* in vitro *activity of chiglitazar in different PPAR subtypes from different cellular contexts, reporter gene-based assays were carried out in the human hepatoma SMMC-7721 and human osteosarcoma U2OS cell lines, and the results were compared with that from other known PPAR agonists. As shown in Table 2, chiglitazar had moderate PPAR*γ* transactivation activity with the EC_50_ values between that of rosiglitazone and pioglitazone, the two marketed TZD-type PPAR*γ* agonists. Chiglitazar also showed significant transactivation in PPAR*α* and PPAR*δ*, which were more potent than that of WY14643 and 2-bromohexadecanoic acid, the known PPAR*α* and PPAR*δ* agonists, respectively. A panel of cell lines with distinct tissue origins and different patterns in expression of individual PPAR subtypes (Figure 1(a)) was treated with chiglitazar and different PPAR agonists, and the expression of selective genes known to be regulated by individual subtypes [4, 6] involved in insulin sensitivity and lipid metabolism was analyzed by RT-PCR. As shown in Figure 1(b), PPAR*γ* downstream genes were upregulated in cell lines treated with both chiglitazar and rosiglitazone, including acyl-CoA oxidase (AOX), hepatic lipase (LIPC), and hormone-sensitive lipase (HSL) in human hepatic SMMC-7721 cells, CD36 in mouse macrophage RAW267.4 cells, and sterol regulatory element binding protein 1 (SREBP1) and adiponectin in mouse preadipocyte 3T3-L1 cells. However, compared with the PPAR*γ* selective agonist rosiglitazone, chiglitazar, as well as WY14643 and 2-bromohexadecanoic acid, more significantly upregulated the expression of PPAR*α* and/or PPAR*δ* downstream genes, including scavenger receptor class A (SRA) in RAW267.4 cells and carnitine palmitoyl transferase 1a and 1b (CPT-1a and CPT-1b) in mouse skeletal muscle C2C12 cells. These *in vitro* results demonstrate that chiglitazar possesses properties as a PPAR pan-agonist. Notably, expression of LDL receptor (LDLR), which is not a previously recognized PPAR target gene, was significantly upregulated by PPAR*δ* agonist 2-bromohexadecanoic acid and downregulated by PPAR*α* or PPAR*γ* agonist WY-14643 and rosiglitazone, while at least kept no change under chiglitazar treatment on SMCC-7721 cells (similar results were also observed on the other human hepatic cell line Bel-7402, data not shown). Given the important role of LDLR in clearance of LDL, this counteractive phenomenon by different subtype-selective agonists, for example, highlighted potentially different impacts on their biological outcome by those subtype-selective or pan-gonists, although the intrinsic mechanism needed to be further elucidated.

### 4.2. *In Vivo* Effects in *db/db* and KKAy Mice

**Figure 2:**
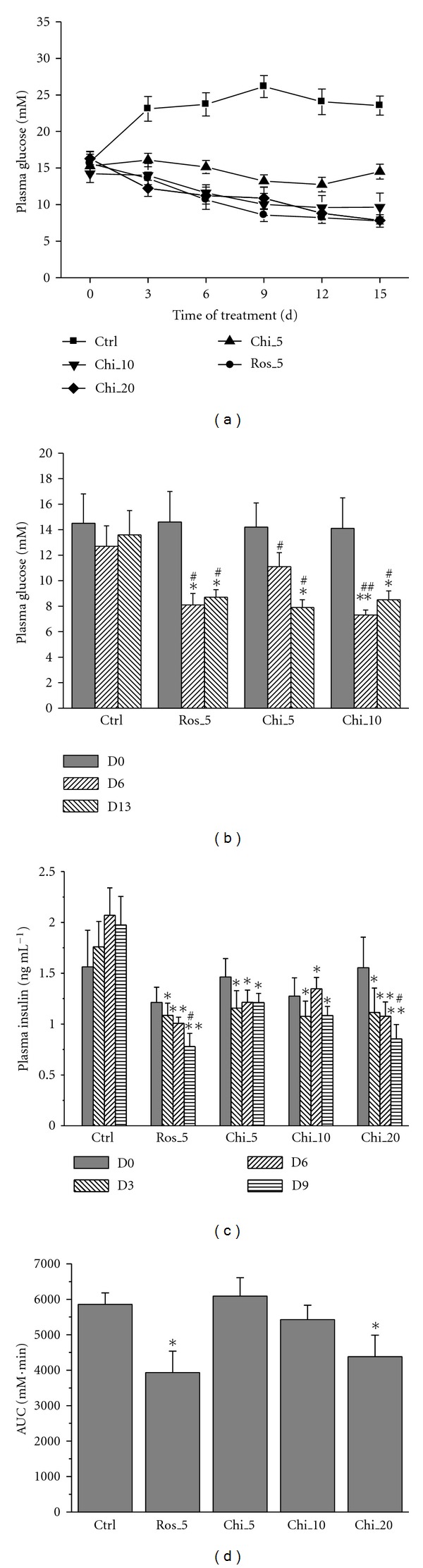
*In vivo* effects on blood glucose control and insulin sensitization of chiglitazar. Male *db/db* (a, c and d) and male KKAy mice (b) were treated with vehicle (Ctrl), rosiglitazone (Ros), or chiglitazar (Chi) at the indicated doses for various days. Plasma glucose (a and b) and insulin (c) levels were measured at the indicated time points. Glucose tolerance (d) was evaluated and the results are presented as the area under curves (AUC) generated from 0, 30, 60, and 120 min after the glucose load. *P < 0.05, and **P < 0.01 compared with Ctrl; ^#^ *P* < 0.05 and ^##^ *P* < 0.01 compared with day 0. *n* = 10 per group for *db/db* mice, and *n* = 8 per group for KKAy mice. Data are expressed as mean ± SE.

**Figure 3:**
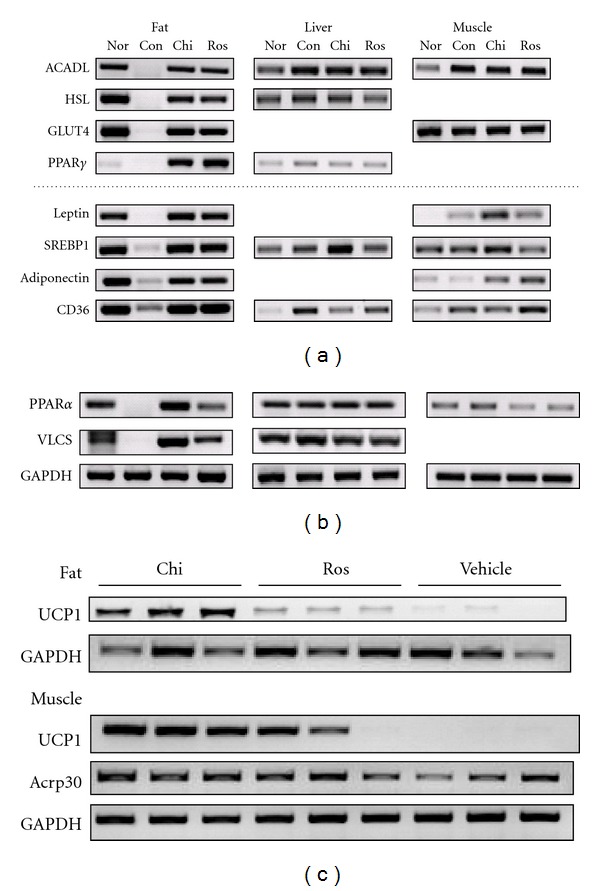
*In vivo* regulation of gene expression by chiglitazar. (a) and (b) Normal male C57BL/6J mice (Nor) and male KKAy mice were orally administered daily with vehicle (Con), chiglitazar of 20 mg kg^−1^ (Chi), or rosiglitazone of 5 mg kg^−1^ (Ros) for 12 days. 24 h after the last dosing, tissues from abdominal fat, liver, and skeletal muscle were taken from 3 mice in each group, and total RNA was isolated from individual animal tissues. Equal amount of total RNA from each mouse in the same group was mixed and subjected to RT-PCR analysis for the indicated genes. (c) Male *db/db* mice were orally administered daily with vehicle (Con), rosiglitazone of 5 mg kg^−1^ (Ros), or chiglitazar of 20 mg kg^−1^ (Chi) for 14 days. 24 h after the last dosing, abdominal fat and skeletal muscle were taken from 3 mice in each group. Total RNA was isolated from individual animal tissues and subjected to the RT-PCR analysis independently for the indicated genes.

**Figure 4:**
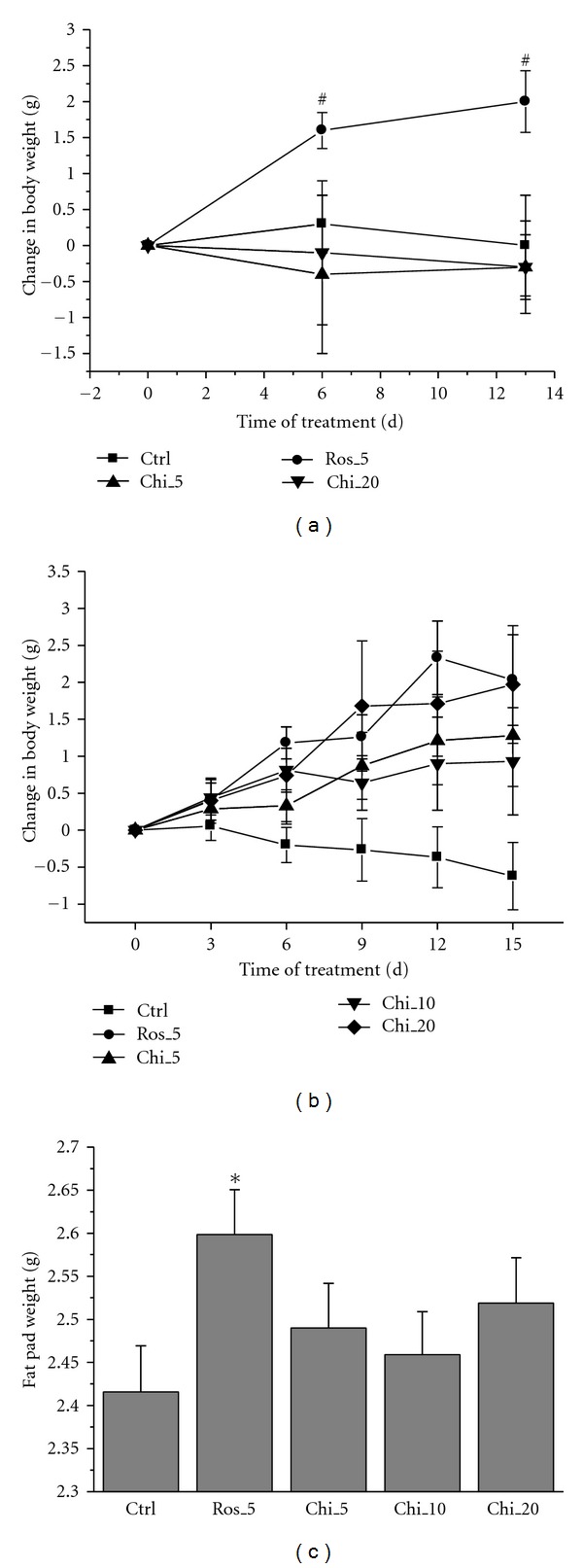
Influence in body and abdominal fat pad weights of chiglitazar. (a) Male KKAy mice were orally administered daily with vehicle (Ctrl), rosiglitazone (Ros) of 5 mg kg^−1^, or chiglitazar (Chi) of 5 and 20 mg kg^−1^ for 12 days, and changes in body weight were evaluated at the indicated days. (b) and (c) Male* db/db* mice were orally administered daily with vehicle (Ctrl), rosiglitazone (Ros) of 5 mg kg^−1^, or chiglitazar (Chi) of 5, 10, and 20 mg kg^−1^ for 14 days. Changes in body weight were evaluated at the indicated days (b), and the fat pads were weighed from the scarified animals at day 15 (c). ^#^ *P* < 0.05 compared with day 0, and *P < 0.05 compared with Ctrl. *n* = 8 per group for KKAy, and *n* = 10 per group for *db/db*. Data are expressed as mean ± SE.

Chiglitazar has previously been shown to significantly improve lipid profile and insulin sensitivity in MSG-induced obese rats [18]. To further evaluate chiglitazar functioning as an insulin sensitizer in diabetic animal models, *db*/*db* and KKAy mice were orally dosed with chiglitazar for a period of 12–14 days and a variety of parameters were examined. Chiglitazar significantly lowered BG levels in *db/db* mice (Figure 2(a)) and KKAy mice (Figure 2(b)) at the dose range between 5 and 20 mg kg^−1^. Compared with the vehicle control group, chiglitazar at different doses also markedly decreased plasma insulin concentrations (Figure 2(c)) and increased the glucose tolerance at the dose of 20 mg kg^−1^ (Figure 2(d)) in *db/db* mice. Adipose tissue, skeletal muscle, and liver were isolated from the animals (adipose tissue only from *db/db *mice) treated with either chiglitazar at 20 mg kg^−1^ or rosiglitazone at 5 mg kg^−1^ (doses at which the two compounds elicited comparable insulin sensitizing effects; Figure 2), and the changes in expression of a panel of genes regulated by the activation of PPARs were analyzed by RT-PCR. As shown in Figure 3(a), genes well known to be involved in insulin sensitization mediated by PPAR*γ* agonists [3] were little expressed in adipose tissue from control KKAy mice, but almost identically up-regulated by the chiglitazar and rosiglitazone treatment. Expression of those genes in liver and skeletal muscle from KKAy mice was either not affected by both compounds (ACADL, HSL, GLUT4 and PPAR*γ*) or more upregulated by chiglitazar (leptin and SREBP1) or by rosiglitazone (Adiponectin and CD36). Meanwhile, compared with rosiglitazone, chiglitazar showed a greater degree of upregulation of PPAR*α* and VLCS in adipose tissue from KKAy mice (Figure 3(b)) and of UCP1 from *db/db *mice (Figure 3(c)). Notably, in comparison with rosiglitazone, chiglitazar did not increase body weight of KKAy mice (Figure 4(a)). Meanwhile, although the induction of body weight increase in *db/db *mice was similar between the treatments with chiglitazar at 20 mg kg^−1^ and rosiglitazone at 5 mg kg^−1^ (Figure 4(b)), less increase in abdominal fat pad weights was observed in chiglitazar-treated animals (Figure 4(c)). 

### 4.3. Influence in Heart Weight and Tissue Distribution in Rats

**Figure 5:**
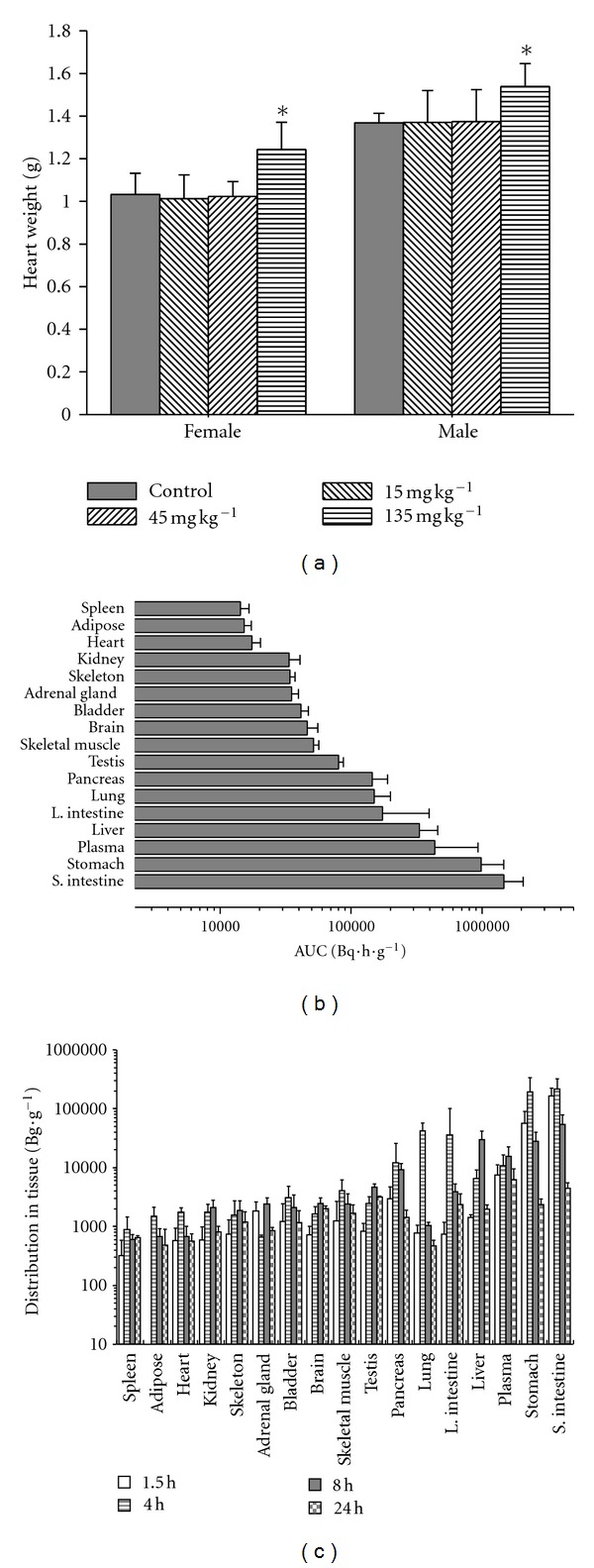
Influence on heart weight and tissue distribution of chiglitazar in rats. (a) Wistar rats were orally administered daily with chiglitazar at the dose of 15, 45 and 135 mg kg^−1^ for 6 months. 24 h after the last dosing, animals were sacrificed and the heart weight was evaluated. *P < 0.05 compared with control; *n* = 5 per group. (b) Male Wistar rats were orally administered with a single dose of ^3^H-chiglitazar at 12.5 mg kg^−1^ (radiation dose 31.7 MBq kg^−1^). Rats were sacrificed at 1.5, 4, 8, and 24 h after treatment (*n* = 5 per time point), and various tissues/organs as indicated were taken for radioactivity measurement. The accumulated area under curves through 0 to 24 h (AUC_0–24_) was calculated based on individual tissue distribution measured at each time point. (c) The time course distribution of chiglitazar in different tissues at individual time point (1.5, 4, 8, 24 h). Data are expressed as mean ± SE.

Cardiac toxicity in animals chronically exposed to PPAR*γ* agonists is a class adverse effect due to PPAR*γ* activation [21]. To evaluate such an adverse effect, Wistar rats were orally dosed with chiglitazar everyday for 6 months, and the changes in heart weight were assessed. As shown in Figure 5(a), chiglitazar did not increase heart weight after 6-month treatment at doses as high as 45 mg kg^−1^ (~300 mg/m^2^), which was about 4.5 times higher than the optimal efficacy dose (10 mg kg^−1^) applied in the Wistar obese rats treated with the compound for 9–40 days [18]. There was about 20% and 12% increase in heart weight at the dose of 135 mg kg^−1^ (~900 mg/m^2^) in female and male rats, respectively (Figure 5(a)). Microscopical examinations revealed that minimal to mild myocardial hypertrophy occurred in the weight-increased hearts at the dose of 135 mg kg^−1^ (data not shown). Chronic treatment of beagle dogs by chiglitazar with the same regime did not induce significant increase in heart weight even at the dose of 54 mg kg^−1^(~1000 mg/m^2^; data not shown). 

H^3^-chiglitazar with single dose of 12.5 mg kg^−1^ (about the optimal efficacy dose) was orally administered to the male Wistar rats, and various organs/tissues were taken at 1.5, 4, 8, and 24 h after the treatment to evaluate the tissue distributions of chiglitazar.  Figure 5(b) shows that high levels of chiglitazar were present in plasma, as well as in liver and pancreas, while low levels were found in kidney, adipose tissue, and heart.

## 5. Discussion

Although the TZD-type PPAR*γ* agonists improve insulin resistance with remarkable efficacy in hyperglycemia, their effects on the associated dyslipidemia are limited. Furthermore, several adverse effects caused by PPAR*γ* activation (i.e., class effects) are well recognized with the use of TZDs in clinical practice, including fluid retention, increased adiposity, and the resulting body weight increase [12, 13]. Two different strategies, which aim at alleviating mechanism-mediated side effects but preserving or improving an overall efficacy profile in T2DM, have been executed in the identification of new generations of synthetic PPAR ligands. One has been to identify PPAR*γ* partial agonists or selective PPAR*γ* modulators to activate only part of the PPAR*γ* transcription-regulatory complex and/or dissociate the insulin sensitizing benefits from the unwanted effects, such as body weight increase [22, 23]. Another has been to, based on the functional importance of individual PPAR subtypes in the regulation of lipid, glucose, and energy homeostasis, discover and develop synthetic ligands to simultaneously target multiple PPAR subtypes (dual or pan-agonists). Although the latter has been hampered by the discontinuations of several PPAR*α*/*γ* dual agonists in clinical development, according to the publicly accessible data, adverse effects of muraglitazar, a PPAR*α*/*γ* dual agonist, appeared to be only linked with the activation of PPAR*γ*, such as fluid retention and increase in body weight [9], as well as the potential cardiovascular safety concerns [10] that have also been raised about rosiglitazone recently [14]. While a synthetic ligand like muraglitazar with the addition of *α* activity did not seem to attenuate *γ* activation-associated adverse effects, it is plausibly based on current knowledge that the additional *δ* activity may contradict some of the unwanted effects caused by the *γ* activation. For example, PPAR*δ* ligands have been shown to retard weight gain in high-fat diet-induced obese animals [7, 24], and produce a favorable lipid profile relevant to the cardiovascular benefits in humans [8, 25]. There is still a solid rationale for the identification of novel synthetic ligands with balanced activating properties in each PPAR subtype and improved *in vivo *profiles. On the other hand, some newly identified non-TZD-type agonists can preserve the glucose lowering effect without concomitant edema and weight gain in animal models by partially stimulating PPAR*γ*-mediated downstream signaling, for example, via differently binding to this receptor and modulating its phosphorylation status at the ser273 site. It is mostly caused by interaction of ligand with previously unrecognized *β*-sheet domain rather than the helix 12 (H12) and AF2 (activation function 2) domain that mediates the classic effect of TZD-type agonists, thus to induce possible different receptor conformation and cofactors recruitment. It further opens the door to separate the significant clinical benefit from those undesired effects with differentiated chemical scaffolds even targeting the troublesome PPAR*γ* [26, 27]. Given the drastic different non-TZD structure nature of chiglitazar and preliminary docking studies with the receptors during the molecule design, it is possible that chiglitazar perhaps also posses other receptor-dependent activity and should be studied in detail in future. 

Chiglitazar has previously been demonstrated to activate PPAR*α* and PPAR*γ* with a much improved lipid profile over rosiglitazone in the MSG-induced obese rats [18]. In the current study we further show that chiglitazar also possesses PPAR*δ* activity, as evidenced by the *in vitro* transactivating reporter gene assays and the RT-PCR analysis in expression of genes regulated by different PPAR subtypes, thereby categorizing the compound as a PPAR pan-agonist. Results from the reporter gene assays suggest that chiglitazar has moderate, but balanced, subtype activities. The knowledge about the correlation between receptor activation properties and overall profiles among different PPAR agonists is very limited, but it is interesting to note from the literature that pioglitazone compared with rosiglitazone is associated with less cardiovascular risks[28] and more significant improvements in lipid profile in T2DM patients [29].Among many possible explanations for an overall better clinical profile shown by pioglitazone is that this compound, compared with rosiglitazone, is less potent in activation of PPAR*γ* and possesses moderate PPAR*α* activating activity as demonstrated by the current report (Table 2) and others [30–33]. Consistent with the results from *in vitro* studies, pioglitazone has been shown to upregulate PPAR*α* and its downstream genes in T2DM patients [34]. Synthetic ligands with moderate, but balanced, activation properties in different PPAR subtypes might eventually prove to be beneficial. 

Our results demonstrate that as a PPAR pan-agonist chiglitazar shows many common features with rosiglitazone functioning as an insulin sensitizer in diabetic *db/db* and KKAy animal models, including the up-regulation of many genes involved in insulin sensitization in adipose tissue, BG-lowering effect, enhanced glucose tolerance, and reduction of plasma insulin. However, differential effects on transcriptional gene regulation were also noticeable between the two compounds from both *in vitro* and *in viv*o studies. First, chiglitazar more significantly up-regulated the expression of PPAR*α* and/or PPAR*δ* downstream genes involved in the key processes of lipid metabolism and thermogenesis [4, 6], including SRA, CPT-1a, and CPT-1b from the investigated cell lines and PPAR*α*, VLCS, and UCP1 in adipose tissue from diabetic mouse models. This differentially regulated gene expression pattern by chiglitazar is quite similar to the profile produced by IL-15 in rodent animals, where IL-15 treatment causes a marked depletion of adipose tissue [35–37] accompanied with the up-regulation of various genes involved in FA oxidation and thermogenesis, including PPAR*δ* and PPAR*α*, UCP-1 and UCP-3, and CPT-1a, and 1b [37]. Our results also show that compared with rosiglitazone, chiglitazar did not significantly increase body weight of KKAy mice and induced less increase in fat pad weights of *db/db* mice. The major difference between two mice models was the function deficiency of leptin receptor (LEPR) in *db/db* mice (LEPR^−^/LEPR^−^), one key cytokine derived from adipose tissue that regulated the food intake and energy expenditure through hypothalamus effects via LEPR to affect fat mass and body weight [38]. In current observation that chiglitazar induced dose-dependent weight gain in *db/db* mice but not in KKAy, perhaps reflected at least partial activity on body weight control by chiglitazar may mediate through Leptin pathway. Whether chiglitazar interfered with leptin signaling to regulate body weight by comprehensive modulation on lipid and energy expenditure needed to be further clarified. Because the assessments of body composition and the functional relevance of differentially regulated genes were not addressed in the current study, further experiments are needed to elucidate the relationship between the gene regulation pattern and gross effects produced by chiglitazar. Second, although both chiglitazar and rosiglitazone up-regulated the expression of SREBP1 in adipose tissue from KKAy mice, only chiglitazar markedly increased the expression of the gene in liver and muscle from the same animal model. SREBP1 is a key transcription factor in regulation of cholesterol and fatty acid metabolism and plays an important role in mediating insulin-dependent actions in various tissues [39]. While it is not known at present if the increased expression of SREBP1 induced by chiglitazar in tissues other than adipose tissue is beneficial, reduced expression of SREBP1 in skeletal muscle as well as adipose tissue was observed in obese and T2DM patients [21, 40], indicating the impaired regulation of the gene in insulin-resistant states. Taken together, the results we observed differentiate chiglitazar from rosiglitazone at both gene and gross body levels, which might prove to be relevant to their overall profiles in patients with T2DM. 

It is known that cardiac toxicity is associated with PPAR*γ* activity from various PPAR agonists both preclinically and clinically. Although it is uncertain if there is direct toxicity to the heart, edema and associated hemodilution induced by the PPAR*γ* agonists, which in turn influence cardiac function, seem to be a class effect [41]. For example, deleting PPAR*γ* from the collecting duct of the nephron eliminates PPAR*γ* ligand-induced weight gain due to water accumulation [42, 43]. In our paper, about 12% and 20% of heart weight increase were observed in male and female rats, respectively, chronically exposed with chiglitazar at the dose of 135 mg kg^−1^ (~900 mg/m^2^) for 6 months, whereas no such an influence was observed at the dose of 45 mg kg^−1^ (~300 mg/m^2^) that was approximately 4.5 times higher than the optimal efficacy dose (10 mg kg^−1^) applied in rats treated with the compound for 9–40 days [18]. Herein, the middle dose (45 mg kg^−1^) was much relevant to the intended clinical dose used in phase III studies (48 mg once daily) by comparing the plasma C_max⁡_ and AUC_0–24hr_ of chiglitazar in rat and human. While the highest dose (135 mg kg^−1^) would be unreachable in current therapeutic application. Chiglitazar had apparently less cardiac toxicity in this regard compared with rosiglitazone and pioglitazone, for which the doses observed to induce heart weight increase in rats for the same length of treatment are 5 and 4 mg kg^−1^, respectively [44, 45]. While it is not known currently whether activation of the *δ* and or *α* subtypes by chiglitazar contributed to the relatively wider therapeutic window between heart weight increase and optimal efficacy observed in rats, it may be that chiglitazar's balanced activation profile combined with its favorable distribution pattern in animals confers a wider therapeutic window. Indeed, chiglitazar was relatively highly distributed in organs/tissues tightly involved in insulin actions (such as liver, pancreas, skeletal muscles) but was only present in low levels in kidney and heart, the 2 critical spots relevant to cardiac toxicity. Of note, chronic treatment of beagle dogs by chiglitazar even at the dose of 54 mg kg^−1^ (~1000 mg/m^2^) for 6 months did not induce significant increase in heart weight (data not shown). Together, these data highlight a safer cardiac profile associated with chiglitazar. 

In summary, the results in the current report reveal the characteristics of chiglitazar as a novel PPAR pan-agonist with *in vitro* and *in vivo* differential effects over the existing PPAR*γ* agonist, rosiglitazone. The results suggest that chiglitazar possesses balanced activity in different PPAR subtypes and a favorable* in vivo* distribution pattern, which might be relevant to its overall encouraging profile in efficacy versus toxicity observed in preclinical animal models, as well as well-tolerated safety profile shown in a completed dose-range clinical studies (phase IIa and IIb) in T2MD patients (manuscript in preparation). 

## Figures and Tables

**Table 1:** Primer sequences used in the RT-PCR analysis.

Gene	Sense	Sequence	GenBank no.
CD36	Forward	ACAGACGCAGCCTCCTTTCC	BC010262
	Reverse	GTCCCAGTCTCATTTAGCCACAG	
SRA	Forward	AAGGTGATCGGGGAGCAATT	NM_138716
	Reverse	CAAAGACAAGAAGAGCAAAAAATTG	
CPT1a	Forward	CCCATGTTGTACAGCTTCCAG	NM_001876
	Reverse	TGGATGGTGTCTGTCTCCTC	
CPT1b	Forward	GCCTTTGTGCAGGCCATGAT	NM_004377
	Reverse	GCTGGGCGTTCGTCTCTGA	
AOX	Forward	CCTGAGCTTCATGCCCTCA	NM_004035
	Reverse	GCCCACTCAAACAAGTTTTCATA	
HSL	Forward	TGCCTGGGCTTCCAGTTCA	NM_005357
	Reverse	GCAGGTCATAGGAGATGAGCCT	
LIPC	Forward	GAACGCACAAGATTGGGAGA	NM_000236.2
	Reverse	GTCTGGGTGATGGCATTGAA	
LDLR	Forward	GATAAGCCTTTCTGGTTTCGG	NM_000527.4
	Reverse	ACATACAACGGGGACATCATTC	
SREBP1	Forward	CTGACAGCTGTGGTGATCCACT	NM_001005291
	Reverse	GCCGGAAGCTCTGTGCCA	
Adiponectin	Forward	GGAGATGCAGGTCTTCTTGGT	NM_009605
	Reverse	TCCTGATACTGGTCGTAGGTGAA	
VLCS	Forward	GGGGCGAAGGTGCTGCT	NM_003645
	Reverse	CCTCGTAAGCCATTTCCCAGT	
UCP1	Forward	ATCACCTTCCCGCTGGACA	NM_021833
	Reverse	TGGCAGGGGACGTCATCTG	
Leptin	Forward	TTCCTGTGGCTTTGGTCCTATC	NM_008493
	Reverse	CACCACCTCTGTGGAGTAGAGTGA	
ACADL	Forward	TCCAAGAAGAAGTGATTCCTCAT	NM_001608
	Reverse	CTGATGAACACCTTGCTTCCAT	
GLUT4	Forward	CAGGTGCTGGGCTTGGAGT	NM_001042
	Reverse	GGCCAGGGCCAATCTCAAA	
PPAR*α*	Forward	GCAAAACTGAAAGCAGAAATTCT	NM_005036
	Reverse	AGCTCCGTGACGGTCTCCA	
PPAR*γ*	Forward	GCCTGCATCTCCACCTTATTATTC	NM_138712
	Reverse	CGCCAACAGCTTCTCCTTCTC	
PPAR*δ*	Forward	GTACTGCCGCTTCCAGAA	NM_006238
	Reverse	GTGCACGCCATACTTGAG	
GAPDH	Forward	ATGCCATCACTGCCACCC	X02231
	Reverse	GCCTGCTTCACCACCTTCTT	
ACTB	Forward	TAGTTGCGTTACACCCTTTC	NM_001101.3
	Reverse	TGTCACCTTCACCGTTCC	
36B4	Forward	CCGTGGTGCTGATGGGCAAGAA	X15267
	Reverse	CCCAAAGCCTGGAAGAAGGA	

**Table 2:** *In vitro *transactivation activity of chiglitazar in different PPAR subtypes.

Compound	PPAR*α*		PPAR*γ*		PPAR*δ*	
	EC_50_ (*μ*M)	% Max^1^	EC_50_ (*μ*M)	% Max^1^	EC_50_ (*μ*M)	% Max^1^
SMMC-7721						

Chi	1.1 ± 0.3	142	0.09 ± 0.02	105	1.7 ± 0.4	123
Ros	8.4 ± 1.5	11	0.04 ± 0.02	100	9.3 ± 2.3	42
Pio	2.2 ± 0.5	130	0.17 ± 0.05	103	4.8 ± 1.2	77
WY	15.5 ± 2.6	100	ia	—	ia	—
2-Bro	ia^2^	—	5.5 ± 1.7	14	5.1 ± 0.9	100

U2OS						

Chi	1.2 ± 0.3	147	0.08 ± 0.02	117	1.5 ± 0.2	244
Ros	7.3 ± 1.9	16	0.04 ± 0.02	100	13.3 ± 3.5	42
Pio	3.2 ± 0.7	152	0.18 ± 0.06	91	4.8 ± 1.3	74
WY	18.0 ± 1.9	100	ia	—	ia	—
2-Bro	ia	—	ia	—	9.2 ± 0.8	100

SMMC-7721 or U2OS cells were transfected individually with the expression plasmids of PPAR*α*, *γ*, or *δ*, together with the reporter constructs containing the corresponding PPAR response elements. hRXR was cotransfected in all experiments. 24 h after transfection, cells were treated with the indicated compounds at various concentrations followed by the measurement of luciferase activity 24 h after treatment. The results shown are the mean values obtained from at least three independent experiments performed in triplicate normalized by the *β*-galactosidase reading. Chi: chiglitazar; Ros: rosiglitazone; Pio: pioglitazone; WY: WY14643; 2-Bro: 2-bromohexadecanoic acid. ^1^% Max response of test compound in transactivation in subtypes *α*, *γ*, and *δ* was compared with that of WY at 50 *μ*M, Ros at 1 *μ*M, and 2-Bro at 10 *μ*M, respectively. ^2^ia: inactive at 10 *μ*M.
